# Elevated C-reactive protein-to-albumin ratio as an independent prognostic marker for mortality in sepsis: a multicenter cohort study

**DOI:** 10.3389/fcimb.2026.1772123

**Published:** 2026-07-08

**Authors:** Yiqu Wei, Wanqing Xu, Shuo Yang, Congfeng Zhang

**Affiliations:** 1 Department of Intensive Care Medicine, Dalian Medical University, Dalian, China; 2 Department of Intensive Care Medicine, Dandong Central Hospital, Dandong, China; 3 Department of Oncology, The First Affiliated Hospital of Zhengzhou University, Zhengzhou, China; 4 Department of Anesthesiology, Dalian Medical University, Dalian, China

**Keywords:** C-reactive protein-to-albumin ratio (CAR), mortality, multicenter cohort study, prognostic biomarker, sepsis

## Abstract

**Background:**

The C-reactive protein-to-albumin ratio (CAR) reflects systemic inflammation and nutritional status, but its prognostic value for mortality in sepsis, particularly across different timeframes, remains uncertain. This multicenter cohort study aimed to assess the association between CAR and all-cause mortality (ACM) in sepsis patients during hospitalization and up to 180 days post-admission.

**Methods:**

We retrospectively analyzed 466 sepsis patients from Dandong Central Hospital, stratified into CAR quartiles (Q1: ≤0.55, Q2: 0.55–3.03, Q3: 3.03–5.96, Q4: >5.96). Baseline characteristics, laboratory results, and clinical outcomes were compared across quartiles and between survivors and non-survivors. Survival was assessed using Kaplan–Meier curves, ROC curves, and Cox regression models with three hierarchical adjustments. External validation was performed with the MIMIC-IV cohort (n = 2,199). Subgroup analyses examined consistency across demographic and clinical subgroups.

**Results:**

Higher CAR was associated with increased mortality at all timepoints: in-hospital (58.6% vs. 38.5%, P = 0.008), 30-day (56.9% vs. 34.2%, P = 0.001), 90-day (58.6% vs. 37.6%, P = 0.007), and 180-day (58.6% vs. 37.6%, P = 0.005). Kaplan-Meier curves showed lower survival in Q4 at all timepoints (P < 0.005), with moderate discriminatory ability (AUC ≈ 0.59). In fully adjusted Cox models, Q4 was independently associated with higher mortality at all time points (HRs: 2.29–2.53, P < 0.01). External validation in MIMIC-IV confirmed similar trends. RCS analyses revealed a linear CAR-mortality relationship (P < 0.001).

**Conclusion:**

Elevated CAR was independently associated with higher mortality in sepsis patients, both during hospitalization and over long-term follow-up, and may serve as a useful prognostic marker when integrated into comprehensive risk-stratification models.

## Introduction

Sepsis is a life-threatening condition resulting from dysregulated host responses to infection, leading to acute organ dysfunction (Singer et al., 2016). It affects over 30 million people globally each year and is responsible for approximately 5 million deaths, accounting for nearly 20% of global mortality (Fleischmann et al., 2016; Rhee et al., 2017; Fleischmann-Struzek et al., 2020). Despite advancements in antimicrobial therapy and organ support, sepsis remains a leading cause of morbidity and mortality in intensive care units (ICUs), with its incidence continuing to rise (Evans et al., 2021; Meyer and Prescott, 2024). Thus, early recognition and accurate risk stratification are crucial for guiding timely interventions, optimizing resource allocation, and improving outcomes in sepsis patients (Zubair et al., 2025).

In recent decades, significant efforts have focused on identifying prognostic biomarkers to assess disease severity and predict mortality (Desautels et al., 2016; Giannini et al., 2019). Due to the complex pathophysiology of sepsis, no single biomarker can reliably capture the full spectrum of immune dysregulation (Ibrahim et al., 2020; Giacobbe et al., 2021). However, combinations of inflammatory and metabolic markers may provide more comprehensive prognostic insights (Huang et al., 2020). Sepsis typically progresses through two immunopathological phases: an initial hyperinflammatory phase, followed by a subsequent immunosuppressive phase. In the early phase, the excessive release of pro-inflammatory mediators triggers cytokine storms and widespread tissue damage (Bloom et al., 2023; Tang et al., 2024; Wang et al., 2024). In contrast, the later phase is characterized by elevated anti-inflammatory cytokines, leading to immune paralysis, secondary infections, and poor outcomes (Rello et al., 2017). These contrasting immune responses highlight the complexity of sepsis and underscore the need for integrative biomarkers that reflect both inflammatory and nutritional status.

C-reactive protein (CRP) is a sensitive and widely used biomarker of systemic inflammation and tissue damage (Rizo-Téllez et al., 2023; Zhou et al., 2024). CRP levels increase rapidly in response to infection and correlate with sepsis severity (Li et al., 2021). However, CRP alone cannot fully predict outcomes, as it primarily reflects the inflammatory burden and does not account for nutritional or metabolic status. Malnutrition, conversely, has become increasingly recognized as a critical determinant of sepsis prognosis. The relationship between inflammation and malnutrition is bidirectional: during sepsis, systemic inflammation reduces appetite, alters protein metabolism, and accelerates catabolism, thereby exacerbating malnutrition (Zhang et al., 2016; Wischmeyer, 2018). In turn, malnutrition impairs immune defenses, increases susceptibility to infections, and exacerbates systemic inflammation (Eckart et al., 2020; Fatyga et al., 2020). Serum albumin (Alb), traditionally considered a nutritional biomarker, is commonly used to assess nutritional status and has prognostic significance across various critical illnesses (Kaysen et al., 2002). However, Alb’s utility is limited, as its levels are influenced by inflammation, fluid balance, and liver function.

To overcome the limitations of single biomarkers, the C-reactive protein-to-albumin ratio (CAR) has emerged as a promising composite index that reflects both systemic inflammation and nutritional reserve. By integrating these two pathophysiological dimensions, CAR may provide a more robust and reliable measure of host vulnerability than CRP or albumin alone. Recent studies have demonstrated the prognostic value of CAR in various clinical contexts. Elevated CAR has been associated with poor outcomes in lung cancer (Deng et al., 2018), ovarian cancer (Liu et al., 2017), esophageal cancer (Wang et al., 2020), liver cancer (Tada et al., 2022; Wang et al., 2024) and critically ill ICU patients (Oh et al., 2018). In these populations, CAR has consistently outperformed traditional single biomarkers, underscoring its potential as an integrative prognostic marker.

Despite these promising findings, evidence regarding the prognostic value of CAR in sepsis remains limited. Given the unique interplay between inflammation and malnutrition in sepsis, CAR could serve as a reliable indicator of disease severity and mortality risk in this patient population. However, most studies have focused on oncology or heterogeneous critical care cohorts, leaving uncertainty about CAR’s predictive value in sepsis-specific contexts. Additionally, the dynamic fluctuations of CRP and albumin during sepsis raise questions about the optimal timing and interpretation of CAR in rapidly evolving disease states.

In this multicenter study, we investigated the prognostic value of CAR in ICU patients with sepsis. Specifically, we aimed to determine whether elevated CAR is independently associated with mortality risk and to assess its performance compared to traditional prognostic indicators. By elucidating the clinical relevance of CAR, we seek to provide clinicians with a simple and readily accessible biomarker to improve early risk stratification and guide individualized management of sepsis.

## Methods

### Study design and data collection

This retrospective cohort study analyzed electronic medical record data from patients with sepsis treated at Dandong Central Hospital between January 2021 and October 2024. Baseline characteristics and laboratory test results obtained within 48 hours of admission were systematically extracted from patient medical records. Blood samples were collected as part of routine clinical care, not prospectively, and corresponding laboratory results were retrieved from the patient records. All samples were processed and analyzed in the hospital’s biochemical laboratory following standard operating procedures. Data were independently collected by two investigators (WYQ and XWQ), and any discrepancies were cross-checked to ensure accuracy and consistency. The study was approved by the Institutional Review Board (IRB) in accordance with the ethical guidelines set forth in the 1964 Declaration of Helsinki. Informed consent was waived because the patient data were fully anonymized.

### Study population

**Figure 1:**
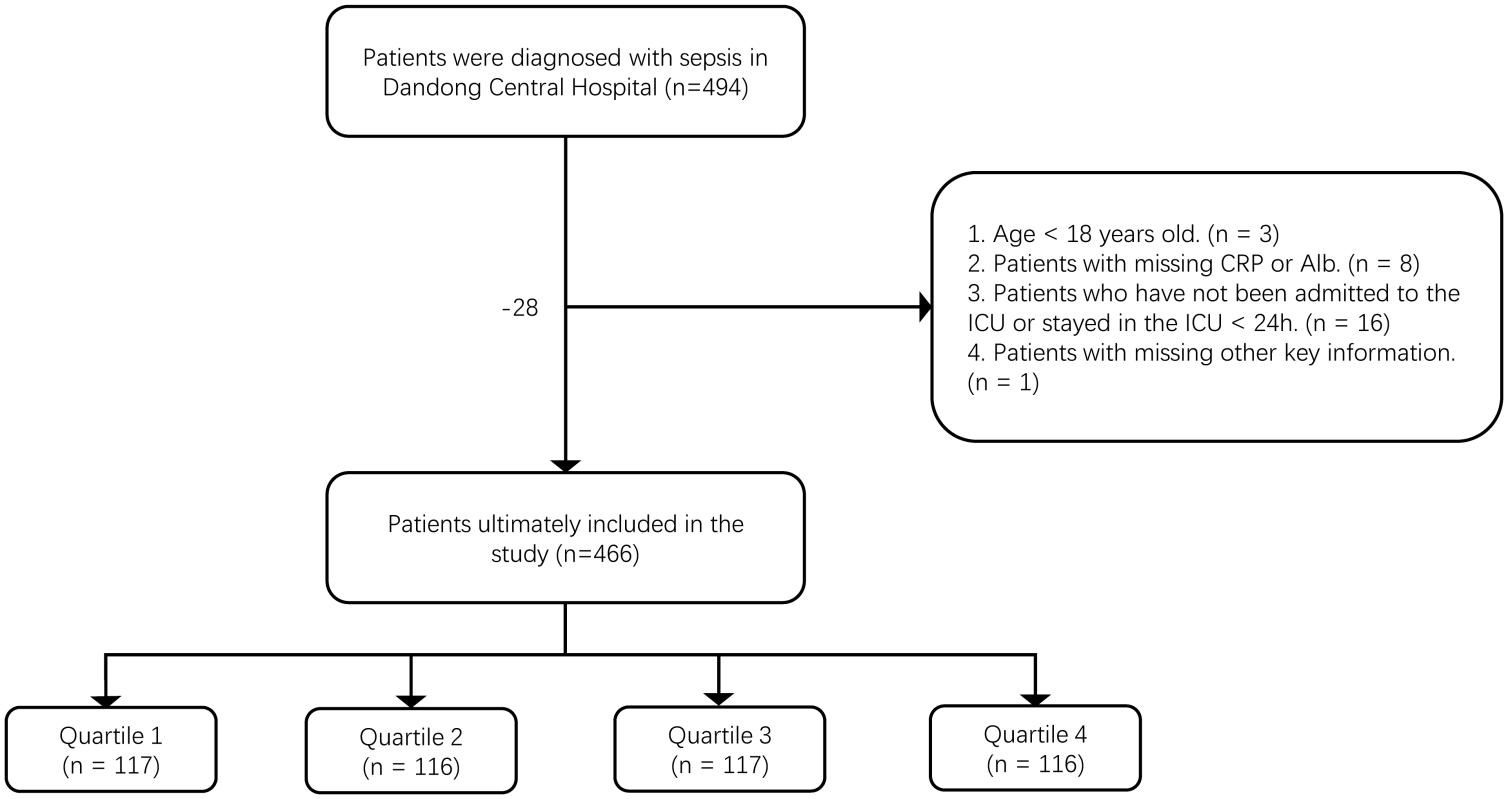
Flowchart of patient selection. A total of 488 sepsis patients were initially screened. Patients were excluded according to the following criteria: (1) age < 18 years (n = 3); (2) missing baseline CRP or albumin values (n = 8); (3) not admitted to the ICU or ICU stay < 24 hours (n = 16); and (4) missing other critical clinical data required for the main analyses, including key laboratory results, vital signs at admission, or essential outcome information (n = 1). The final internal cohort consisted of 466 patients.

The study population consisted of patients diagnosed with sepsis. The inclusion criteria were as follows: (1) age ≥ 18 years; and (2) fulfillment of the Sepsis-3 diagnostic criteria. The exclusion criteria were: (1) age < 18 years; (2) missing C-reactive protein (CRP) or albumin (Alb) data; (3) patients who were not admitted to the ICU or who stayed in the ICU for less than 24 hours; and (4) missing other key information. A total of 466 patients met the inclusion criteria and were categorized into different groups based on the C-reactive protein-to-albumin ratio (CAR) index: Q1 (CAR ≤ 0.55, N = 117), Q2 (0.55 < CAR ≤ 3.03, N = 116), Q3 (3.03 < CAR ≤ 5.96, N = 117), and Q4 (CAR > 5.96, N = 116) (**Figure 1**).

### Validation cohort based on the MIMIC-IV database

To externally validate our findings, we conducted a supplementary analysis using data from the publicly available Medical Information Mart for Intensive Care IV (MIMIC-IV, version 3.1) database. MIMIC-IV contains de-identified clinical information from patients admitted to the Beth Israel Deaconess Medical Center (Boston, MA, USA) between 2008 and 2022. We included adult patients (≥18 years) diagnosed with sepsis, while excluding those with multiple admissions, ICU stays shorter than 24 hours, or missing data on C-reactive protein (CRP) or albumin (Alb). A total of 2,199 patients met the inclusion criteria and were stratified into quartiles based on their CAR levels: Q1 (CAR ≤ 12.02), Q2 (12.02 < CAR ≤ 30.04), Q3 (30.04 < CAR ≤ 60.15), and Q4 (CAR > 60.15) (**Supplementary Figure 1**). All data in MIMIC-IV are fully anonymized, and thus informed consent was waived. Access to the database and analysis were approved by the Institutional Review Board of the Massachusetts Institute of Technology (certification number: 58407754).

### Exposure

The primary exposure variable in both cohorts was the C-reactive protein-to-albumin ratio (CAR), which integrates indicators of systemic inflammation and nutritional status. CAR was calculated by dividing C-reactive protein (CRP) (mg/L) by albumin (Alb) (g/L) and analyzed both as a continuous variable and by quartiles. The CRP and albumin levels used for CAR calculation were the first available values within this 48-hour window.

In the Dandong Central Hospital cohort, patients were categorized into four CAR quartiles: Q1 (CAR ≤ 0.55, n = 117), Q2 (0.55 < CAR ≤ 3.03, n = 116), Q3 (3.03 < CAR ≤ 5.96, n = 117), and Q4 (CAR > 5.96, n = 116).

In the MIMIC-IV validation cohort, quartile cutoffs were determined based on the distribution of CAR in this population: Q1 (CAR ≤ 12.02, n = 550), Q2 (12.02 < CAR ≤ 30.04, n = 550), Q3 (30.04 < CAR ≤ 60.15, n = 549), and Q4 (CAR > 60.15, n = 550).

Low albumin levels indicate nutrient depletion, while elevated CRP levels reflect inflammation. Together, these markers capture the combined effects of nutrient depletion and systemic inflammation, both of which are critical factors influencing the outcomes of patients with sepsis.

### Laboratory measurements

Serum albumin and C-reactive protein were measured in the hospital laboratory using standard automated biochemical and immunoturbidimetric assays, respectively. The normal reference range for serum albumin was 35–55 g/L, while CRP was typically considered elevated at values >10 mg/L (values <10 mg/L are generally within the normal range in healthy individuals). In the MIMIC-IV database, CRP and albumin measurements were obtained from routine clinical laboratory testing using hospital-standardized platforms. Although differences in analytical platforms and reagents may exist between institutions, both datasets reflect clinically validated measurements commonly used in critical care practice.

### Outcome

The primary outcome was in-hospital all-cause mortality (ACM). Secondary outcomes varied slightly between cohorts due to differences in data availability. For the Dandong cohort, secondary outcomes included 30-day, 90-day, and 180-day ACM. For the MIMIC-IV cohort, secondary outcomes included 30-day and 180-day ACM following hospital admission.

### Statistical analysis

All analyses were conducted separately for the Dandong and MIMIC-IV cohorts, followed by a qualitative comparison of the effect estimates. Continuous variables were tested for normality. Normally distributed data are presented as mean ± standard deviation (SD) and were compared using Student’s t-test or one-way ANOVA. Non-normally distributed data are presented as medians with interquartile ranges (IQR) and were compared using the Wilcoxon rank-sum test. Categorical variables are expressed as counts and percentages and were analyzed using the Chi-square test or Fisher’s exact test.

Kaplan–Meier (K–M) curves and log-rank tests were used to assess survival differences across CAR quartiles. Univariable and multivariable Cox proportional hazards regression models were constructed to examine the association between CAR and mortality.

For the Dandong cohort,

- Model 1 was unadjusted;
- Model 2 adjusted for age and gender;
- Model 3 further adjusted for hypertension, diabetes, lung disease, heart disease, cerebrovascular disease, renal disease, and a range of physiological and biochemical parameters (heart rate [HR], respiratory rate [RR], systolic blood pressure [SBP], temperature, lymphocytes, monocytes, hematocrit, platelets, thrombocytocrit, potassium, creatinine, glucose, uric acid, alkaline phosphatase, total cholesterol, HDL, APTT, TT, FDP, D-dimer, procalcitonin [PCT], NT-proBNP, hs-cTnI, PO_2_, and HbA1c).

For the MIMIC-IV validation cohort,

- Model 1 was unadjusted;
- Model 2 adjusted for age, gender, and BMI;
- Model 3 further adjusted for myocardial infarction, congestive heart failure, cerebrovascular disease, chronic pulmonary disease, hypertension, diabetes, and key laboratory parameters (HR, RR, SBP, diastolic blood pressure [DBP], temperature, SpO_2_, white blood cell count [WBC], hemoglobin [Hb], platelets, blood urea nitrogen [BUN], calcium, hematocrit, creatinine, sodium, potassium, anion gap, bicarbonate, chloride, international normalized ratio [INR], prothrombin time [PT], and APTT).

In both datasets, the lowest CAR quartile served as the reference group. Additionally, CAR was modeled as a continuous variable using restricted cubic splines (RCS) to explore potential non-linear associations with mortality risk.

Subgroup analyses were stratified by demographic and clinical characteristics:

- Dandong cohort: age, gender, hypertension, diabetes, lung disease, heart disease, cerebrovascular disease, and renal disease;
- MIMIC-IV cohort: age, gender, myocardial infarction, congestive heart failure, cerebrovascular disease, chronic pulmonary disease, diabetes, and hypertension.

All statistical analyses were performed using R software (version 4.3.2; R Foundation for Statistical Computing, Vienna, Austria), and a two-sided P value < 0.05 was considered statistically significant.

## Results

### Baseline characteristics of study individuals

Stratification by CAR quartiles revealed significant heterogeneity in baseline characteristics, laboratory indices, and clinical outcomes across the combined cohort (**Table 1**; **Supplementary Table 1**). Higher CAR levels were consistently associated with more advanced systemic inflammation and nutritional depletion, as reflected by progressively elevated CRP and decreased Alb concentrations (both P < 0.001). Patients in the highest CAR quartile exhibited greater physiological instability, characterized by higher HR and RR, lower BP, and reduced SpO_2_ (all P < 0.05). Laboratory findings indicated more severe organ dysfunction, including elevated creatinine, urea, and inflammatory cell counts, alongside coagulopathy with prolonged PT, APTT, and elevated INR. Lipid abnormalities, such as hypertriglyceridemia and reduced HDL cholesterol, were also prominent. Clinical severity scores, such as the SOFA score, increased steadily with higher CAR levels (P < 0.001). Higher CAR quartiles were significantly associated with greater illness severity as measured by the SOFA score (P < 0.001). Mortality demonstrated a clear dose–response relationship, with the highest CAR quartile showing significantly higher in-hospital and long-term mortality across both cohorts (all P < 0.01). Notably, absolute CAR values differed substantially between the two cohorts, reflecting differences in laboratory distributions and patient characteristics; however, the graded association between higher CAR quartiles and increased disease severity and mortality was consistent across both populations. These findings collectively highlight CAR as a composite biomarker that integrates inflammatory and nutritional stress, strongly correlating with disease severity and poor prognosis in sepsis patients.

**Table 1:** Baseline characteristics of patients grouped according to CAR index quartiles.

Variables	Total (N=466)	Q1 (N=117, ≤0.55)	Q2 (N=116, 0.55-3.03)	Q3 (N=117, 3.03-5.96)	Q4 (N=116, >5.96)	P value*
Female, (%)	209 (44.8%)	49 (41.9%)	51 (44.0%)	55 (47.0%)	54 (46.6%)	0.849
Age, year	70.7 ± 13.6	68.4 ± 15.2	71.7 ± 13.2	72.0 ± 12.3	70.7 ± 13.4	0.505
Complication						
Hypertension, (%)	217 (46.6%)	55 (47.0%)	53 (45.7%)	56 (47.9%)	53 (45.7%)	0.984
Diabetes, (%)	161 (34.5%)	38 (32.5%)	34 (29.3%)	45 (38.5%)	44 (37.9%)	0.379
Lung diseases, (%)	82 (17.6%)	17 (14.5%)	24 (20.7%)	19 (16.2%)	22 (19.0%)	0.619
Heart disease, (%)	145 (31.1%)	48 (41.0%)	37 (31.9%)	29 (24.8%)	31 (26.7%)	0.039
Cerebrovascular diseases, (%)	156 (33.5%)	34 (29.1%)	39 (33.6%)	39 (33.3%)	44 (37.9%)	0.552
Renal disease, (%)	78 (16.7%)	25 (21.4%)	23 (19.8%)	20 (17.1%)	10 (8.6%)	0.048
Vital signs						
HR, beats/min	102.4 ± 24.7	102.4 ± 21.6	100.3 ± 27.5	99.6 ± 23.5	107.4 ± 25.4	0.086
RR, times/min	19.3 ± 2.8	19.3 ± 3.0	19.5 ± 2.5	19.0 ± 2.6	19.3 ± 3.0	0.522
SBP, mmHg	110.1 ± 28.4	114.4 ± 30.9	112.7 ± 27.0	110.0 ± 27.0	103.2 ± 27.6	0.016
DBP, mmHg	66.8 ± 17.1	69.7 ± 19.0	67.6 ± 14.8	66.4 ± 15.4	63.5 ± 18.4	0.023
Temp, °C	37.1 ± 1.0	37.3 ± 1.1	37.1 ± 1.0	37.0 ± 1.0	36.9 ± 1.0	0.016
SpO2, %	90.6 ± 8.6	91.6 ± 7.8	90.7 ± 8.4	90.2 ± 9.5	90.0 ± 8.8	0.689
Laboratory parameters						
WBC, 10^9/L	13.6 ± 7.6	12.7 ± 7.2	12.9 ± 7.3	15.2 ± 7.6	13.4 ± 8.1	0.041
RBC, 10^9/L	3.9 ± 0.9	3.9 ± 1.0	4.1 ± 0.8	3.9 ± 0.8	3.8 ± 0.9	0.246
Hb, g/L	118.5 ± 28.1	118.8 ± 32.0	122.2 ± 27.1	118.9 ± 25.9	114.2 ± 27.0	0.362
Platelets, 10^9/L	181.7 ± 102.6	200.6 ± 103.9	191.4 ± 97.4	179.4 ± 98.3	155.2 ± 106.0	0.002
Potassium, mmol/L	4.0 ± 0.7	4.0 ± 0.7	3.9 ± 0.7	4.0 ± 0.8	3.9 ± 0.8	0.746
Sodium, mmol/L	137.5 ± 4.9	137.7 ± 4.8	137.3 ± 4.8	137.7 ± 4.9	137.3 ± 5.1	0.872
Chlorine, mmol/L	102.3 ± 5.4	101.9 ± 5.5	101.7 ± 5.4	102.5 ± 5.4	103.1 ± 5.1	0.301
Urea, mmol/L	12.8 ± 7.3	11.6 ± 6.6	11.8 ± 6.7	13.5 ± 8.1	14.4 ± 7.3	0.006
Creatinine, umol/L	152.8 ± 97.0	130.5 ± 83.4	138.9 ± 90.0	170.9 ± 105.6	171.0 ± 101.6	0.001
Glucose, mmol/L	8.5 ± 3.8	7.9 ± 3.4	8.3 ± 3.5	8.6 ± 4.0	9.1 ± 4.1	0.105
ALT, U/L	34.8 ± 22.4	34.8 ± 22.3	36.5 ± 23.8	34.6 ± 21.9	33.2 ± 21.8	0.701
AST, U/L	48.5 ± 36.1	48.2 ± 36.8	50.1 ± 38.4	47.1 ± 34.9	48.5 ± 34.7	0.992
Albumin, g/L	32.0 ± 7.0	33.6 ± 7.8	33.9 ± 6.1	32.3 ± 6.0	28.3 ± 6.4	<0.001
Triglycerides, mmol/L	1.6 ± 0.8	1.3 ± 0.6	1.4 ± 0.7	1.8 ± 0.9	1.9 ± 0.9	<0.001
HDL, mmol/L	0.8 ± 0.4	0.9 ± 0.4	0.9 ± 0.4	0.8 ± 0.4	0.6 ± 0.3	<0.001
LDL, mmol/L	2.1 ± 0.8	2.2 ± 0.8	2.1 ± 0.8	2.1 ± 0.8	2.0 ± 0.7	0.298
PT, s	15.3 ± 2.4	15.0 ± 2.6	14.9 ± 2.4	15.4 ± 2.2	15.9 ± 2.4	<0.001
APTT, s	33.0 ± 6.8	31.2 ± 6.0	31.0 ± 5.9	33.8 ± 6.7	36.1 ± 7.4	<0.001
TT, s	15.4 ± 2.0	15.1 ± 2.0	15.2 ± 1.9	15.7 ± 1.9	15.5 ± 2.0	0.035
INR	1.2 ± 0.2	1.2 ± 0.2	1.2 ± 0.2	1.2 ± 0.2	1.3 ± 0.2	0.001
FDP, ug/ml	19.3 ± 17.9	17.5 ± 16.9	19.3 ± 18.8	20.5 ± 18.8	20.1 ± 17.2	0.339
Blood gas analysis						
PH	7.4 ± 0.1	7.4 ± 0.1	7.4 ± 0.1	7.4 ± 0.1	7.4 ± 0.1	0.204
PCO2, mmHg	31.4 ± 8.1	31.8 ± 8.8	30.9 ± 7.7	32.0 ± 7.8	30.9 ± 8.2	0.514
PO2, mmHg	94.0 ± 36.0	89.7 ± 36.1	95.7 ± 35.6	96.6 ± 37.3	94.0 ± 35.1	0.617
SB, mmol/L	19.5 ± 4.9	19.0 ± 4.8	19.3 ± 5.1	19.9 ± 5.0	19.8 ± 4.7	0.382
BE, mol/L	-6.4 ± 6.4	-7.0 ± 6.0	-7.0 ± 6.8	-5.8 ± 6.4	-5.8 ± 6.3	0.259
AG, mmol/L	14.6 ± 4.8	14.5 ± 5.2	14.8 ± 4.7	13.9 ± 4.6	15.1 ± 4.6	0.188
Lac, mmol/L	4.0 ± 2.7	4.3 ± 2.9	4.1 ± 2.7	3.5 ± 2.5	4.1 ± 2.8	0.235
FiO2, %	37.8 ± 12.5	35.8 ± 11.6	36.1 ± 12.7	37.4 ± 12.0	42.0 ± 12.9	<0.001
Length of stay						
Los in Hospital, day	12.3 ± 13.6	14.4 ± 19.0	12.1 ± 11.7	11.7 ± 11.1	10.8 ± 10.5	0.125
Los in ICU, day	6.2 ± 6.4	6.2 ± 5.7	5.3 ± 5.7	6.1 ± 5.8	7.1 ± 8.0	0.230
Outcomes						
In-hospital ACM, (%)	208 (44.6%)	45 (38.5%)	48 (41.4%)	47 (40.2%)	68 (58.6%)	0.008
30-Day ACM, (%)	195 (41.8%)	40 (34.2%)	46 (39.7%)	43 (36.8%)	66 (56.9%)	0.001
90-Day ACM, (%)	207 (44.4%)	44 (37.6%)	48 (41.4%)	47 (40.2%)	68 (58.6%)	0.007
180-Day ACM, (%)	207 (44.4%)	44 (37.6%)	48 (41.4%)	47 (40.2%)	68 (58.6%)	0.005

*Statistically significant: a value less than 0.05 is interpreted as a meaningful difference.

CAR, C-reactive protein to albumin ratio; HR, Heart rate; RR, Respiratory rate; SBP, Systolic blood pressure; DBP, Diastolic blood pressure; Temp, Temperature; SpO2, Peripheral capillary oxygen saturation; WBC, White blood cells; RBC, Red blood cells; Hb, Hemoglobin; ALT, Alanine aminotransferase; AST, Aspartate aminotransferase; HDL, High-density lipoprotein; LDL, Low-density lipoprotein; PT, Prothrombin time; APTT, Activated partial thromboplastin time; TT, Thrombin time; INR, International normalized ratio; FDP, Fibrin Degradation Products; PH, Potential of hydrogen; PCO2, Partial Pressure of Carbon Dioxide; PO2, Partial Pressure of Oxygen; SB, Standard Bicarbonate; BE, Base Excess; AG, Anion Gap; Lac, Lactic Acid; FiO2, Fraction of Inspired Oxygen; LOS, Length of Stay; ICU, Intensive care unit; ACM, All-Cause Mortality.

A comparison between survivors and non-survivors revealed pronounced pathophysiological differences (**Table 2**; **Supplementary Table 2**). Non-survivors were generally older, had higher CAR levels, and exhibited more severe hypoalbuminemia and systemic inflammation (all P < 0.001). They also had a heavier burden of comorbidities, including cardiovascular and pulmonary diseases, and demonstrated hemodynamic instability, characterized by tachycardia, hypotension, and hypoxemia. Laboratory analyses showed evidence of multiorgan dysfunction—elevated creatinine and urea, metabolic acidosis with an increased anion gap and lactate, and pronounced coagulopathy with prolonged PT, APTT, and higher INR values (all P < 0.01). Nutritional and hepatic stress indicators, such as lower HDL, higher triglycerides, and elevated liver enzymes, were also more prevalent among non-survivors. Consistently across both cohorts, these patients had higher illness severity scores, longer ICU stays, and significantly higher short- and long-term mortality, defining a phenotype characterized by systemic inflammation, metabolic derangement, and multiorgan failure, which predicted poor outcomes in sepsis.

**Table 2:** Baseline characteristics of patients grouped based on in-hospital survival status.

Variables	Total (N=466)	Survival (N=258)	Non-Survival (N=208)	P value*
Female, (%)	209 (44.8%)	115 (44.6%)	94 (45.2%)	0.925
Age, year	70.7 ± 13.6	68.0 ± 14.1	74.0 ± 12.2	<0.001
Complication				
Hypertension, (%)	217 (46.6%)	120 (46.5%)	97 (46.6%)	1.000
Diabetes, (%)	161 (34.5%)	89 (34.5%)	72 (34.6%)	1.000
Lung diseases, (%)	82 (17.6%)	33 (12.8%)	49 (23.6%)	0.003
Heart disease, (%)	145 (31.1%)	68 (26.4%)	77 (37.0%)	0.022
Cerebrovascular diseases, (%)	156 (33.5%)	86 (33.3%)	70 (33.7%)	1.000
Renal disease, (%)	78 (16.7%)	40 (15.5%)	38 (18.3%)	0.447
Vital signs				
HR, beats/min	102.4 ± 24.7	99.3 ± 22.4	106.2 ± 26.8	0.003
RR, times/min	19.3 ± 2.8	19.1 ± 2.2	19.5 ± 3.3	0.272
SBP, mmHg	110.1 ± 28.4	111.0 ± 27.2	108.9 ± 29.8	0.337
DBP, mmHg	66.8 ± 17.1	68.4 ± 16.5	64.8 ± 17.7	0.027
Temp, °C	37.1 ± 1.0	37.1 ± 1.0	37.0 ± 1.0	0.022
SpO2, %	90.6 ± 8.6	92.2 ± 8.1	88.6 ± 8.9	<0.001
Laboratory parameters				
WBC, 10^9/L	13.6 ± 7.6	13.3 ± 7.4	13.9 ± 7.9	0.354
RBC, 10^9/L	3.9 ± 0.9	4.0 ± 0.8	3.8 ± 1.0	0.012
Hb, g/L	118.5 ± 28.1	121.1 ± 25.3	115.3 ± 31.1	0.026
Platelets, 10^9/L	181.7 ± 102.6	181.3 ± 98.3	182.1 ± 107.9	0.941
Potassium, mmol/L	4.0 ± 0.7	3.9 ± 0.7	4.1 ± 0.8	0.028
Sodium, mmol/L	137.5 ± 4.9	137.2 ± 4.4	137.9 ± 5.4	0.228
Chlorine, mmol/L	102.3 ± 5.4	102.1 ± 5.0	102.6 ± 5.8	0.374
Urea, mmol/L	12.8 ± 7.3	10.7 ± 6.1	15.5 ± 7.7	<0.001
Creatinine, umol/L	152.8 ± 97.0	130.9 ± 84.5	180.0 ± 104.7	<0.001
Glucose, mmol/L	8.5 ± 3.8	8.2 ± 3.6	8.8 ± 4.0	0.097
ALT, U/L	34.8 ± 22.4	33.6 ± 21.2	36.2 ± 23.8	0.511
AST, U/L	48.5 ± 36.1	43.0 ± 31.2	55.2 ± 40.5	0.001
Albumin, g/L	32.0 ± 7.0	33.5 ± 6.6	30.2 ± 7.1	<0.001
Triglycerides, mmol/L	1.6 ± 0.8	1.5 ± 0.8	1.7 ± 0.8	0.004
HDL, mmol/L	0.8 ± 0.4	0.8 ± 0.4	0.7 ± 0.3	0.004
LDL, mmol/L	2.1 ± 0.8	2.0 ± 0.7	2.2 ± 0.8	0.024
PT, s	15.3 ± 2.4	15.0 ± 2.3	15.7 ± 2.6	0.004
APTT, s	33.0 ± 6.8	32.6 ± 6.0	33.5 ± 7.7	0.397
TT, s	15.4 ± 2.0	15.0 ± 1.8	15.8 ± 2.0	<0.001
INR	1.2 ± 0.2	1.2 ± 0.2	1.3 ± 0.2	0.004
FDP, ug/ml	19.3 ± 17.9	18.2 ± 18.5	20.8 ± 17.1	0.004
Blood gas analysis				
PH	7.4 ± 0.1	7.4 ± 0.1	7.4 ± 0.1	0.002
PCO2, mmHg	31.4 ± 8.1	32.2 ± 7.8	30.4 ± 8.4	0.017
PO2, mmHg	94.0 ± 36.0	93.2 ± 35.2	95.1 ± 37.1	0.731
SB, mmol/L	19.5 ± 4.9	20.6 ± 4.3	18.2 ± 5.3	<0.001
BE, mol/L	-6.4 ± 6.4	-5.1 ± 5.7	-8.0 ± 6.8	<0.001
AG, mmol/L	14.6 ± 4.8	13.5 ± 4.4	15.8 ± 5.1	<0.001
Lac, mmol/L	4.0 ± 2.7	3.5 ± 2.3	4.6 ± 3.1	<0.001
FiO2, %	37.8 ± 12.5	36.1 ± 11.8	40.0 ± 13.0	<0.001
Length of stay				
Los in Hospital, day	12.3 ± 13.6	14.3 ± 10.9	9.7 ± 15.9	<0.001
Los in ICU, day	6.2 ± 6.4	5.3 ± 5.6	7.3 ± 7.1	0.010

*Statistically significant: a value less than 0.05 is interpreted as a meaningful difference.

CAR, C-reactive protein to albumin ratio; HR, Heart rate; RR, Respiratory rate; SBP, Systolic blood pressure; DBP, Diastolic blood pressure; Temp, Temperature; SpO2, Peripheral capillary oxygen saturation; WBC, White blood cells; RBC, Red blood cells; Hb, Hemoglobin; ALT, Alanine aminotransferase; AST, Aspartate aminotransferase; HDL, High-density lipoprotein; LDL, Low-density lipoprotein; PT, Prothrombin time; APTT, Activated partial thromboplastin time; TT, Thrombin time; INR, International normalized ratio; FDP, Fibrin Degradation Products; PH, Potential of hydrogen; PCO2, Partial Pressure of Carbon Dioxide; PO2, Partial Pressure of Oxygen; SB, Standard Bicarbonate; BE, Base Excess; AG, Anion Gap; Lac, Lactic Acid; FiO2, Fraction of Inspired Oxygen; LOS, Length of Stay; ICU, Intensive care unit; ACM, All-Cause Mortality.

### Survival analysis

**Figure 2:**
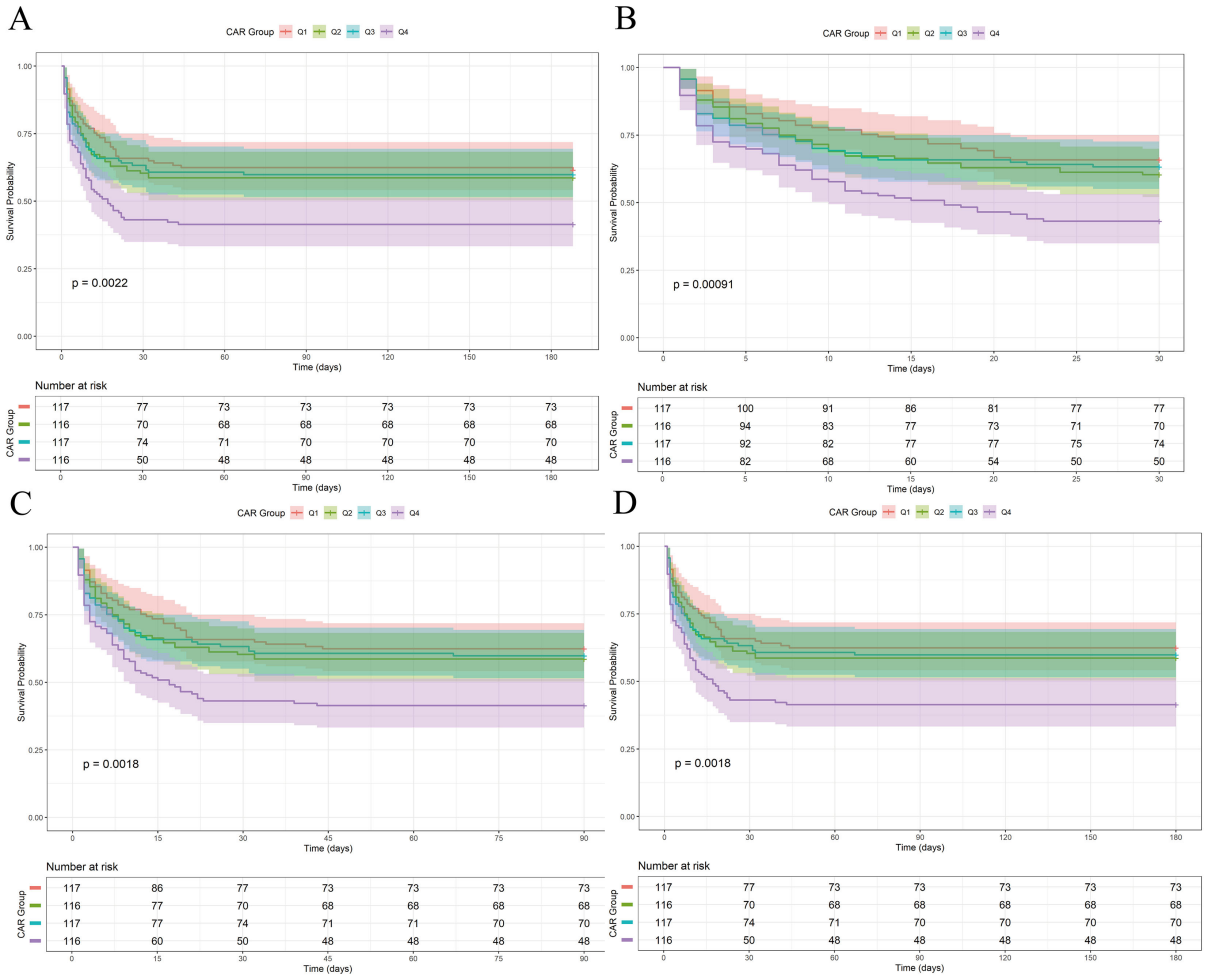
Kaplan-Meier survival analysis curves for all-cause mortality. **(A)** In-hospital mortality; **(B)** 30-day mortality; **(C)** 90-day mortality; **(D)** 180-day mortality.

Kaplan–Meier survival curves revealed a significant decline in survival probability with increasing CAR quartiles (**Figure 2**). Patients in the highest CAR quartile (Q4) exhibited consistently lower survival rates compared to those in Q1 at all time points, with significant differences observed in in-hospital (p = 0.002), 30-day (p = 0.001), 90-day (p = 0.002), and 180-day mortality (p = 0.002). The separation of survival curves began early during hospitalization and persisted throughout follow-up, showing a clear dose–response gradient (Q1 > Q2 > Q3 > Q4). The number of event-free patients declined most rapidly in Q4, indicating markedly higher early and mid-term mortality risks. These findings suggest that elevated CAR levels are strongly associated with poor survival outcomes, supporting CAR as an independent prognostic marker in sepsis.

**Figure 3:**
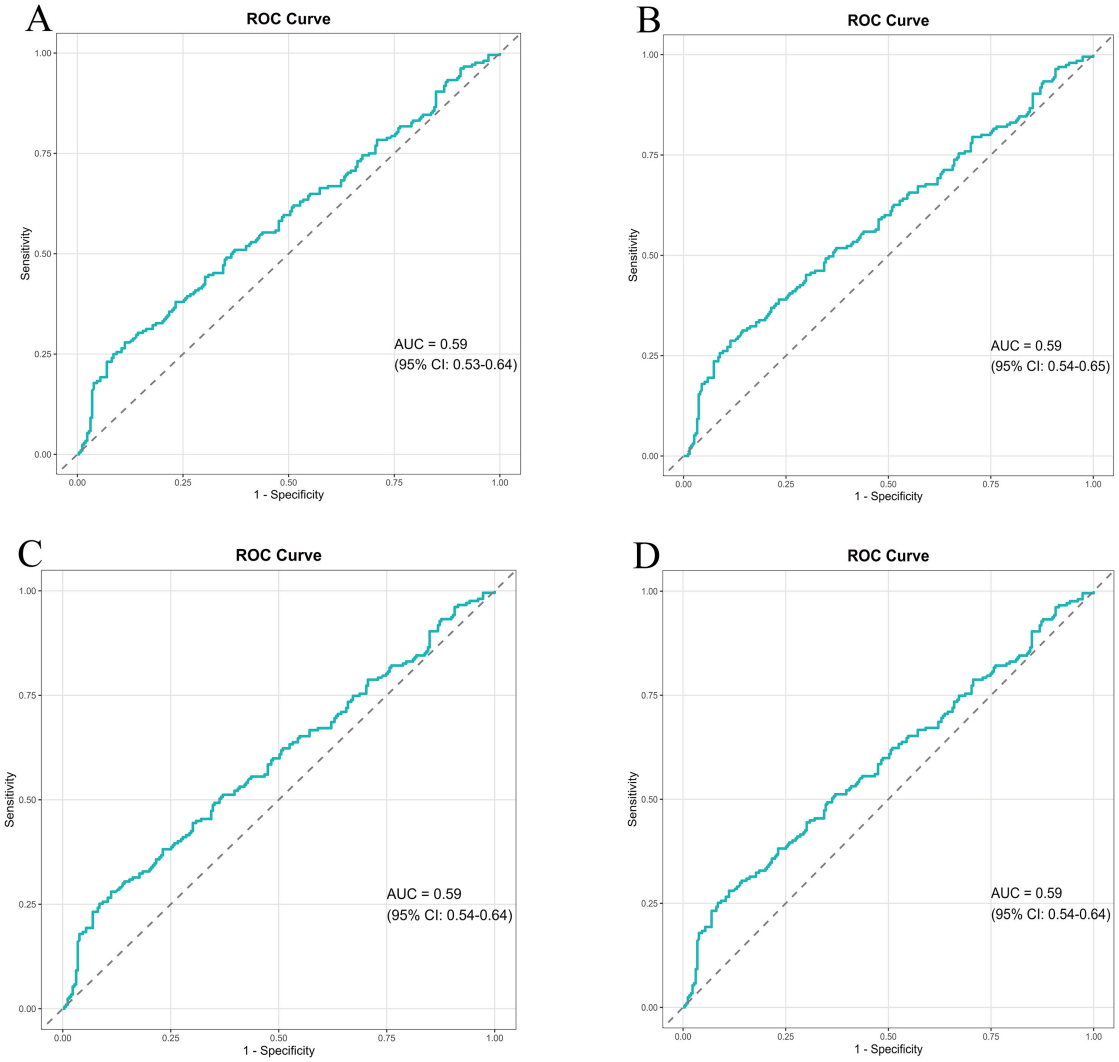
Receiver operating characteristic (ROC) curve analysis. **(A)** In-hospital mortality; **(B)** 30-day mortality; **(C)** 90-day mortality; **(D)** 180-day mortality.

Receiver operating characteristic (ROC) analyses were conducted to assess the discriminative performance of CAR for predicting mortality (**Figure 3**). The area under the curve (AUC) values were consistent across time points: 0.59 for in-hospital mortality (95% CI: 0.53–0.64), 0.59 for 30-day mortality (95% CI: 0.54–0.65), 0.59 for 90-day mortality (95% CI: 0.54–0.64), and 0.59 for 180-day mortality (95% CI: 0.54–0.64). These values indicate that CAR has a consistent but modest predictive ability. The ROC curves demonstrated reasonable specificity at low false-positive rates, suggesting that CAR effectively identifies a meaningful proportion of high-risk patients. Although its standalone discriminative power is moderate, the temporal stability of the AUCs supports CAR as a reliable biomarker for inclusion in multivariable risk models for mortality prediction in sepsis.

The area under the curve (AUC) values were consistently ~0.59 across all time points (95% CI: 0.53–0.65), indicating fair discriminative ability as a prognostic marker. While modest, this performance is comparable to several other single or composite inflammatory/nutritional markers reported in the sepsis literature (Ranzani et al., 2013; Kılınç Toker et al., 2024; Pan et al., 2025) and supports the inclusion of CAR in multivariable risk assessment.

### Comparison of CAR with the SOFA score in the MIMIC-IV cohort

To further explore the incremental prognostic value of CAR relative to an established severity score, we performed a supplementary ROC analysis in the MIMIC-IV validation cohort, where SOFA scores are comprehensively recorded. The AUC for in-hospital mortality using the SOFA score alone was 0.65 (95% CI: 0.62–0.68). When CAR was added to the SOFA score, the AUC increased modestly to 0.66 (95% CI: 0.63–0.69) (**Supplementary Figures 2**, **S3**). These results suggest a small incremental improvement in discriminative performance; however, formal reclassification metrics were not calculated in the present study. Due to the absence of complete SOFA data in the internal Dandong Central Hospital cohort, this comparison could not be performed in the derivation set.

### Correlation between CAR and outcomes

In both the internal and external cohorts, higher CAR levels were independently associated with an increased risk of mortality across multiple timeframes (**Table 3**). In the Dandong Central Hospital cohort, a clear stepwise increase in mortality was observed across CAR quartiles. The highest quartile (Q4, CAR > 5.96) demonstrated significantly elevated risks for in-hospital (HR = 2.29, 95% CI: 1.40–3.74; P = 0.002), 30-day (HR = 2.53, 95% CI: 1.52–4.22; P = 0.003), and 90-day mortality (HR = 2.36, 95% CI: 1.44–3.86; P = 0.019) after full multivariable adjustment. These associations remained robust across all hierarchical models, with significant P-for-trend values (P < 0.05). Consistent results were observed in the MIMIC-IV validation cohort, where patients in the highest CAR quartile (Q4 ≥ 60.15) showed significantly increased risks for in-hospital (HR = 1.38, 95% CI: 1.06–1.79; P = 0.016), 30-day (HR = 1.55, 95% CI: 1.18–2.04; P = 0.002), and 180-day mortality (HR = 1.42, 95% CI: 1.16–1.75; P = 0.001) compared with Q1 (**Supplementary Table 3**). The consistent, graded pattern of risk across both cohorts supports CAR as a stable and independent prognostic biomarker in sepsis. Importantly, the strong independent association between higher CAR quartiles and mortality persisted even after full adjustment for multiple laboratory parameters, indicating robustness to potential overadjustment.

**Table 3:** Multivariate Cox proportional hazards analysis of CAR quartiles and mortality.

Categories	Model1		Model2		Model3	
	HR (95%CI)	P value	HR (95%CI)	P value	HR (95%CI)	P value
In-Hospital ACM						
Quartile*						
Q1	Ref		Ref		Ref	
Q2	1.13 (0.75-1.70)	<0.001	1.05 (0.70-1.57)	<0.001	1.21 (0.78-1.89)	<0.001
Q3	1.11 (0.74-1.67)	<0.001	1.07 (0.71-1.61)	<0.001	0.90 (0.57-1.43)	0.001
Q4	1.88 (1.29-2.74)	<0.001	1.86 (1.27-2.71)	<0.001	2.29 (1.40-3.74)	0.002
P for trend		0.002		0.002		0.009
30-Day ACM						
Quartile						
Q1	Ref		Ref		Ref	
Q2	1.22 (0.80-1.86)	<0.001	1.13 (0.74-1.73)	<0.001	1.36 (0.85-2.17)	<0.001
Q3	1.14 (0.74-1.76)	<0.001	1.10 (0.72-1.69)	0.001	0.91 (0.56-1.49)	0.001
Q4	2.02 (1.37-3.00)	<0.001	2.00 (1.35-2.97)	0.003	2.53 (1.52-4.22)	0.003
P for trend		<0.001		<0.001		0.005
90-Day ACM						
Quartile						
Q1	Ref		Ref		Ref	
Q2	1.16 (0.77-1.74)	0.001	1.07 (0.71-1.61)	0.025	1.25 (0.80-1.95)	0.044
Q3	1.14 (0.75-1.71)	<0.001	1.09 (0.72-1.65)	0.004	0.93 (0.58-1.48)	0.006
Q4	1.92 (1.31-2.81)	<0.001	1.90 (1.30-2.78)	0.008	2.36 (1.44-3.86)	0.019
P for trend		0.001		0.001		0.006
180-Day ACM						
Quartile						
Q1	Ref		Ref		Ref	
Q2	1.16 (0.77-1.74)	0.002	1.07 (0.71-1.61)	0.053	1.25 (0.80-1.95)	0.093
Q3	1.14 (0.75-1.71)	<0.001	1.09 (0.72-1.65)	0.006	0.93 (0.58-1.48)	0.011
Q4	1.92 (1.31-2.81)	<0.001	1.90 (1.30-2.78)	0.021	2.36 (1.44-3.86)	0.075
P for trend		0.001		0.001		0.006

Model 1: no adjustment.

Model 2: adjusted for Age, Gender.

Model 3: further adjusted for Age, Gender, Hypertension, Diabetes, Lung diseases, Heart disease, Cerebrovascular diseases, Renal disease, HR, RR, SBP, Temp, Lymphocytes, Monocytes, Hematocrit, Platelets, Thrombocytocrit, Potassium, Creatinine, Glucose, UA, Alkaline, TC, HDL, APTT, TT, FDP, D-Dimer, PCT, NT-proBNP, Hs-cTnI, PO2, HbA1c.

*CAR Quartile: Q1 (≤0.55), Q2 (0.55-3.03), Q3 (3.03-5.96), Q4 (>5.96).

**Figure 4:**
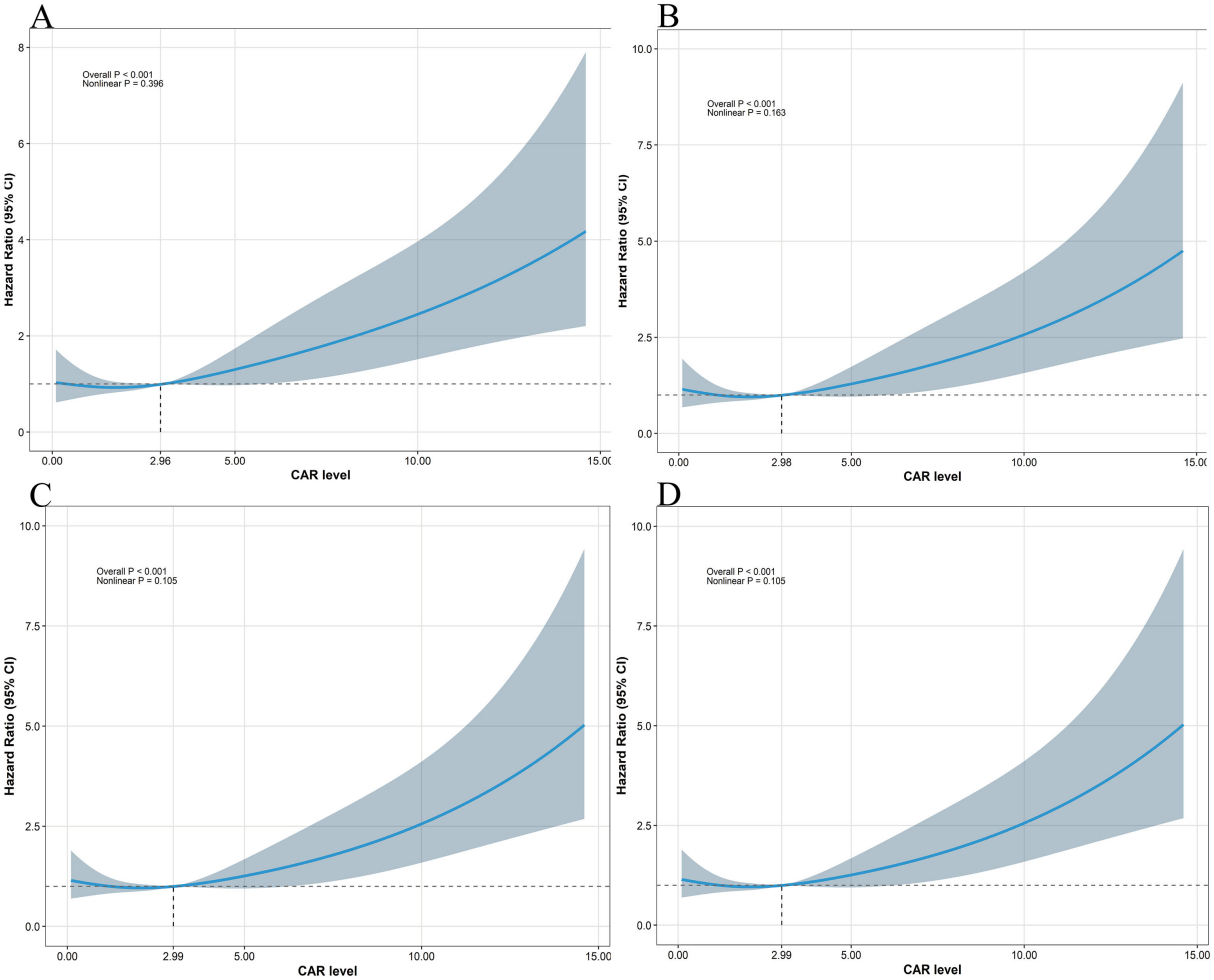
Restricted cubic spline (RCS) analysis demonstrating the linear dose–response relationship between CAR and all-cause mortality in **(A)** In-hospital mortality, **(B)** 30-day mortality, **(C)** 90-day mortality, and **(D)** 180-day mortality. The shaded areas represent 95% confidence intervals.

RCS modeling in the hospital cohort revealed a linear dose–response relationship between CAR and mortality (**Figure 4**). Mortality risk remained lowest at CAR values around 3.0 and increased steadily thereafter. The overall associations were statistically significant (all P < 0.001), with no evidence of nonlinearity (P > 0.05). Hazard ratios rose approximately four- to fivefold at CAR values above 15.0, and the lower confidence limits consistently exceeded unity for CAR > 5.0, indicating a persistent adverse prognostic effect. The nearly identical curves across in-hospital, 30-day, 90-day, and 180-day analyses further suggest the temporal stability of this association. Collectively, these findings confirm that CAR is linearly and independently associated with mortality risk, reinforcing its clinical utility as a quantitative and biologically plausible prognostic indicator.

### Subgroup analyses

**Figure 5:**
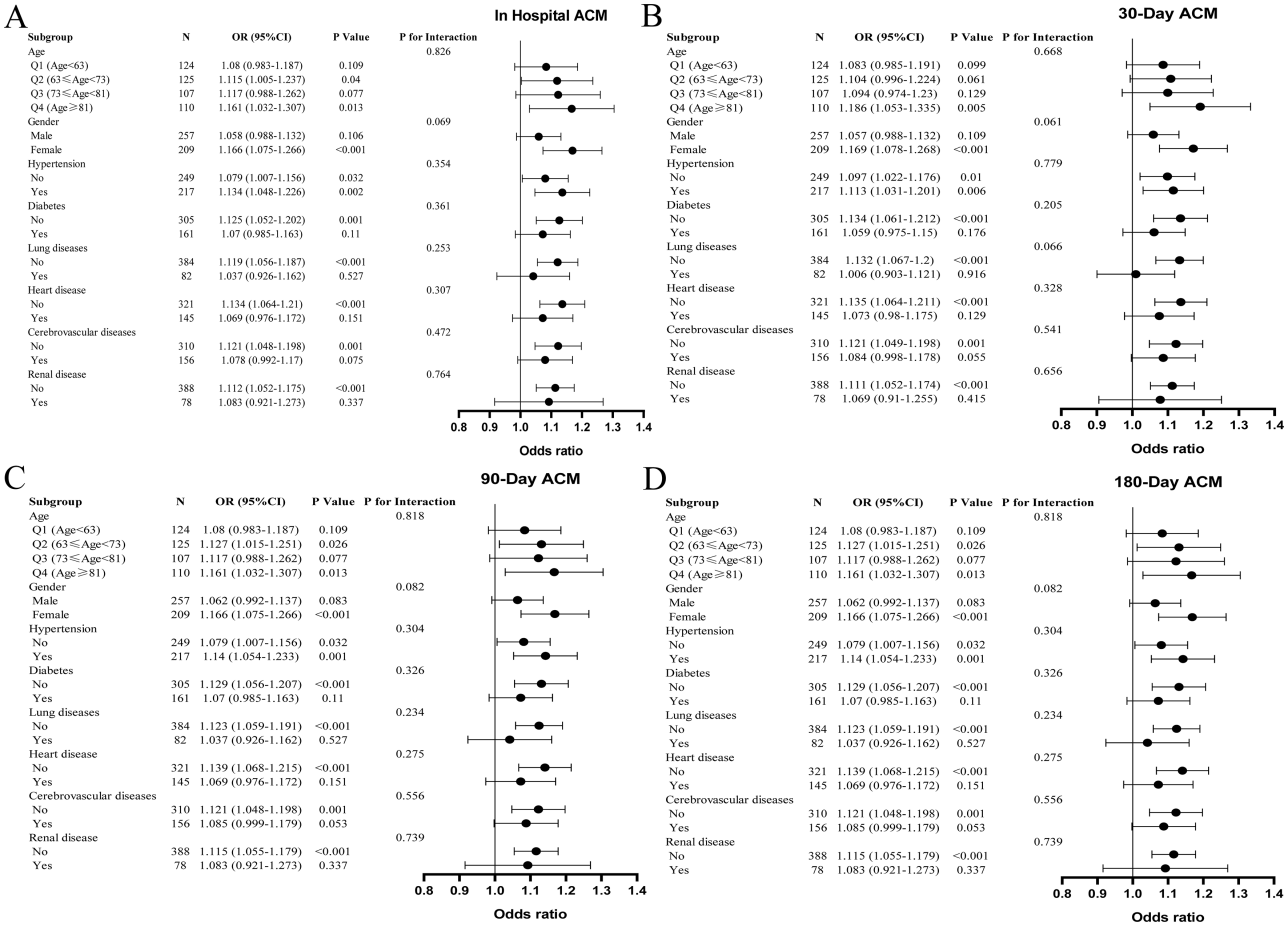
Forest plots illustrating stratified analyses of association of CAR and all-cause mortality. **(A)** In-hospital mortality; **(B)** 30-day mortality; **(C)** 90-day mortality; **(D)** 180-day mortality.

Subgroup analyses from both the internal and external cohorts demonstrated a broadly consistent and independent association between elevated CAR levels and increased mortality risk across all predefined strata (**Figure 5**). The positive relationship between CAR and mortality was observed across demographic and clinical subgroups, including age, gender, and major comorbidities, with no significant interaction effects detected (all P-interaction > 0.05). In both cohorts, the association was stronger among females, older adults, and patients with hypertension or cardiovascular disease, while attenuated or non-significant associations were seen in those with chronic pulmonary or renal diseases, suggesting potential effect modification by organ-specific comorbidities.

In the MIMIC-IV validation cohort, similar trends were observed, with higher CAR values consistently predicting increased mortality across most subgroups, including those without myocardial infarction or diabetes (**Supplementary Figure 4**). Importantly, the absence of significant interactions across both datasets underscores the robustness and generalizability of CAR as a prognostic biomarker. Collectively, these findings suggest that the predictive effect of CAR on mortality is largely independent of traditional risk factors and remains stable across diverse patient subpopulations, while certain chronic comorbid conditions may partially modulate its discriminative strength.

## Discussion

In this multicenter cohort study, which included 466 patients from our hospital and 2,199 patients from the MIMIC-IV database, elevated CAR was independently associated with higher short- and long-term mortality in sepsis. Patients in the highest CAR quartile consistently exhibited significantly increased risks of in-hospital, 30-day, 90-day, and 180-day mortality across both cohorts, even after adjusting for demographics, comorbidities, vital signs, and a comprehensive array of laboratory indices. Restricted cubic spline analysis confirmed a primarily linear, dose-dependent relationship between CAR and mortality, indicating that the risk progressively increases with higher CAR levels. Subgroup analyses further reinforced the robustness of these associations across various strata, including age, gender, and major comorbidities. This multicenter cohort study demonstrates that elevated CAR is independently associated with higher short- and long-term mortality in sepsis and qualifies as a prognostic marker.

Sepsis is a critical medical condition characterized by life-threatening organ dysfunction due to an uncontrolled and dysregulated host response to infection (Rudd et al., 2020). The underlying mechanisms involve a complex network of systemic inflammation, immune dysregulation, endothelial damage, and significant metabolic disturbances (Grondman et al., 2020). During the initial hyperinflammatory phase, excessive secretion of proinflammatory cytokines such as IL-6, TNF-α, and IL-1β leads to endothelial activation, increased vascular permeability, impaired microcirculatory flow, and compromised oxygen delivery to tissues, ultimately resulting in multiorgan failure (de Pádua Lúcio et al., 2018; Vural et al., 2023; Vural et al., 2024). At the same time, compensatory anti-inflammatory responses can induce immune paralysis, making patients susceptible to secondary infections and prolonged recovery (Wischmeyer, 2018). Metabolic abnormalities are also common in sepsis, including protein-calorie malnutrition, decreased serum albumin levels, and dysregulated lipid metabolism, all of which exacerbate immune dysfunction and delay tissue regeneration (McLaughlin et al., 2020). Together, these interconnected mechanisms highlight the multifactorial complexity of sepsis pathogenesis, which cannot be fully captured by a single biomarker.

Given this complex biological landscape, composite biomarkers that integrate both systemic inflammation and nutritional status may more accurately reflect the overall disease burden and predict outcomes better than isolated markers. CRP, a positive acute-phase reactant, rapidly increases in response to inflammation and infection, indicating both the magnitude and duration of the inflammatory response (Li et al., 2021). In contrast, serum albumin (Alb), a negative acute-phase protein, tends to decrease due to increased vascular permeability, capillary leak, hepatic reprioritization of protein synthesis, and suppressed hepatic synthesis during acute illness. This opposing dynamic results in a strong intrinsic inverse correlation between CRP and albumin levels in sepsis and other critical illnesses (Don and Kaysen, 2004). By combining these two mechanistically complementary markers, the C-reactive protein-to-albumin ratio (CAR) amplifies the signal of systemic inflammation and nutritional derangement, providing a more comprehensive and sensitive measure of both inflammatory burden and physiological reserve than either parameter alone.

Furthermore, elevated CAR may reflect broader pathophysiological processes central to sepsis progression, including endothelial dysfunction, oxidative stress, and microvascular injury, which contribute to multiple organ dysfunction syndrome (MODS) (Joffre and Hellman, 2021). Hypoalbuminemia exacerbates fluid shifts and impairs oncotic pressure, while sustained high CRP levels correlate with persistent cytokine storm and immune dysregulation (Wiedermann, 2021). These mechanisms collectively link higher CAR values to increased risk of hemodynamic instability, prolonged ICU stay, and higher mortality across short- and long-term follow-up periods. Our findings of a linear relationship on restricted cubic spline analysis further support the dose-response nature of this association. Future studies integrating CAR with biomarkers of endothelial injury (e.g., angiopoietin-2) or immune function may further elucidate these pathways (Joffre and Hellman, 2021).

Emerging evidence consistently supports the prognostic value of CAR in sepsis. A meta-analysis involving 3,224 patients demonstrated that higher CAR levels were significantly associated with increased mortality risk (pooled HR = 1.10; AUC = 0.82) (Magoon, 2021). Subsequent ICU studies showed that CAR outperformed either CRP or Alb alone in mortality prediction, with an AUC of 0.843 compared to 0.820 and 0.813, respectively (Oh et al., 2018). Furthermore, CAR assessed at ICU discharge was a strong predictor of 90-day mortality (Ranzani et al., 2013; Weber et al., 2020). Prognostic models incorporating CAR from the MIMIC-IV database achieved high predictive performance (C-index ≈ 0.92–0.94; AUC ≈ 0.80–0.88) (Matsubara et al., 2023). Additional cohort studies and systematic reviews have further confirmed that CAR enhances risk stratification and demonstrates superior prognostic performance compared to traditional biomarkers (Nagri et al., 2023; Zhang et al., 2024). Taken together, these findings suggest that an elevated CAR reflects sustained systemic inflammation (high CRP) and diminished nutritional or immune reserves (low Alb), functioning as a synergistic indicator of vulnerability. This “dual impact” mechanism may worsen organ dysfunction, delay recovery, and ultimately reduce both short- and long-term survival in sepsis patients.

This study has several limitations. First, the retrospective design of both the hospital and MIMIC-IV cohorts limits causal inference. Despite comprehensive adjustments, residual confounding from unmeasured variables cannot be excluded. Second, the internal cohort was derived from a single center in China, and the MIMIC-IV cohort predominantly represents U.S. populations. These differences in ethnicity, case mix, sepsis etiology, and healthcare systems may limit the generalizability of our findings to other regions and populations. Although external validation in MIMIC-IV partially strengthens the robustness of the associations, further large-scale, multicenter, international studies are still needed to confirm the prognostic utility of CAR across diverse clinical settings. Third, the mechanistic pathways linking elevated CAR to adverse outcomes were not directly investigated. Prospective, multicenter studies are needed to validate these findings, elucidate underlying biological mechanisms, and optimize CAR-based risk stratification for sepsis. Fourth, CAR was calculated based on a single early measurement within 48 hours of admission and therefore does not reflect the dynamic changes in inflammatory and nutritional status during the course of sepsis. In addition, due to the retrospective nature of this study and limitations in available diagnostic coding, we were unable to systematically exclude patients with conditions that may independently alter CRP or albumin levels, such as active autoimmune diseases or rare congenital inflammatory disorders. These conditions may introduce residual confounding in CAR measurement. Although we adjusted for a wide range of comorbidities and laboratory parameters in multivariable models, unmeasured confounding cannot be fully excluded. Future prospective studies with more granular clinical phenotyping are warranted to address this issue.

In addition, The AUC value of approximately 0.59 for CAR in predicting mortality reflects only fair discriminative performance when used in isolation. This is consistent with previous reports of composite inflammatory markers in heterogeneous sepsis cohorts, where standalone AUC values frequently range from 0.55 to 0.70. Although CAR qualifies as an independent prognostic marker in multivariable models, its moderate discriminative ability underscores that it should not be viewed as a standalone replacement for validated clinical scores but rather as a complementary, easily obtainable tool to enhance early risk identification, particularly in resource-limited settings or when combined with existing severity scoring systems.

## Conclusion

In this multicenter cohort study, elevated CAR was independently associated with higher mortality in sepsis patients, both in the short and long term. The association was consistent, predominantly linear, and robust across most subgroups. As a readily available biomarker, CAR may assist in early risk stratification and prognostic assessment in sepsis.

## Data Availability

The raw data supporting the conclusions of this article will be made available by the authors, without undue reservation.

## Supplementary material

Supplementary Figure 1 Flowchart of select study population from the MIMIC-IV database.

Supplementary Figure 2 ROC curve of the SOFA score alone for predicting in-hospital mortality in the MIMIC-IV validation cohort.

Supplementary Figure 3 ROC curve of the combination of the SOFA score and the CAR for predicting in-hospital mortality in the MIMIC-IV validation cohort.

Supplementary Figure 4 Forest plots illustrating stratified analyses of association of CAR and all-cause mortality. **(A)** In-Hospital Mortality; **(B)** 30-Day Mortality; **(C)** 180-Day Mortality.

Supplementary Table 1 Baseline characteristics and outcomes of participants classified by CAR quartiles.

Supplementary Table 2 Baseline characteristics of patients grouped based on In-Hospital Survival Status.

Supplementary Table 3 Multivariate Cox Proportional Hazards Analysis of CAR Quartiles and Mortality.
