# Supplementary figures and images for: Elevated C-reactive protein-to-albumin ratio as an independent prognostic marker for mortality in sepsis: a multicenter cohort study
Source: Front Cell Infect Microbiol. 2026 Jul 8;16:1772123. doi: 10.3389/fcimb.2026.1772123 (PMC13388168; doi:10.3389/fcimb.2026.1772123)

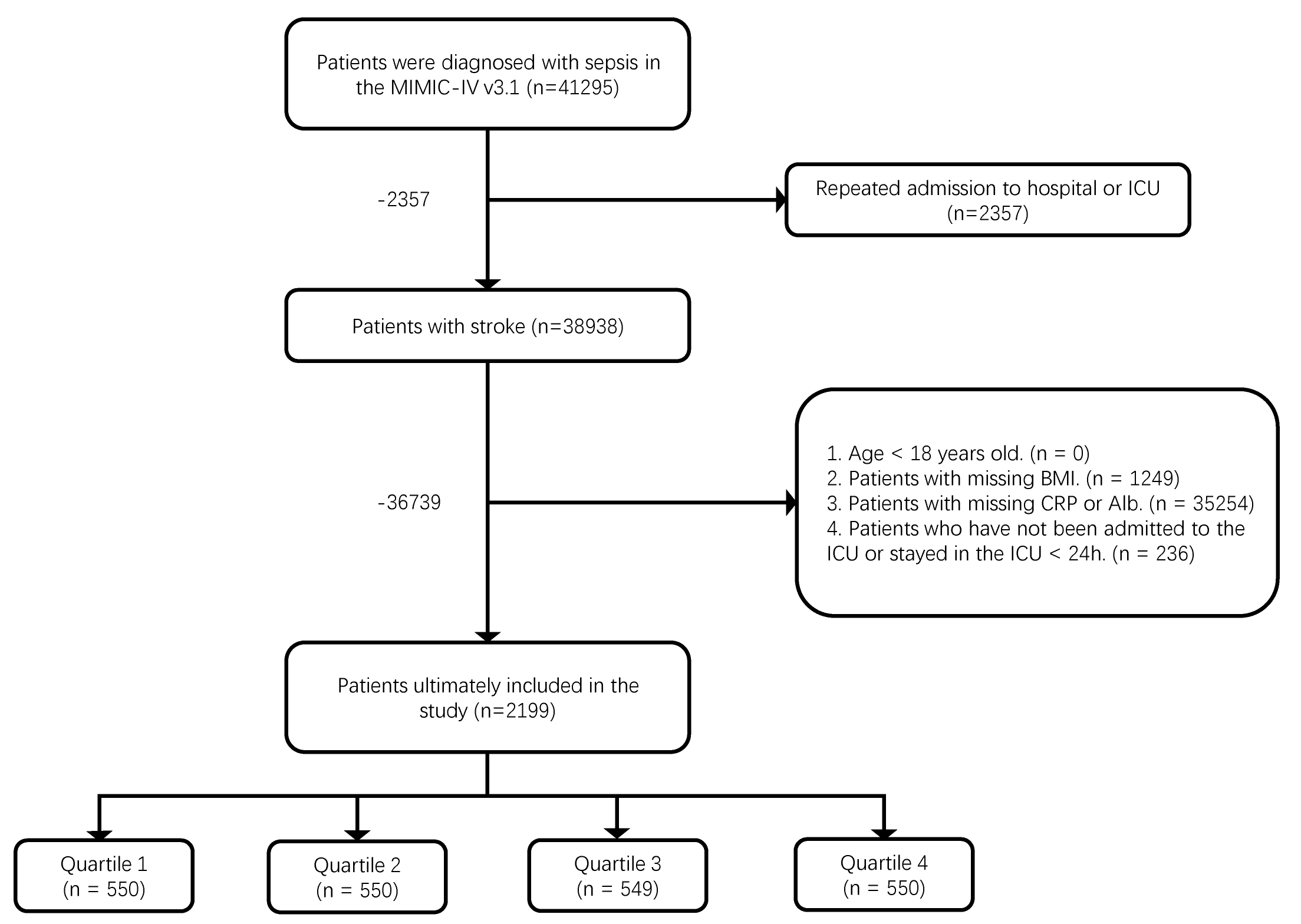

Supplement: Supplementary Figure 1 — Flowchart of select study population from the MIMIC-IV database. [file Image1.tif]

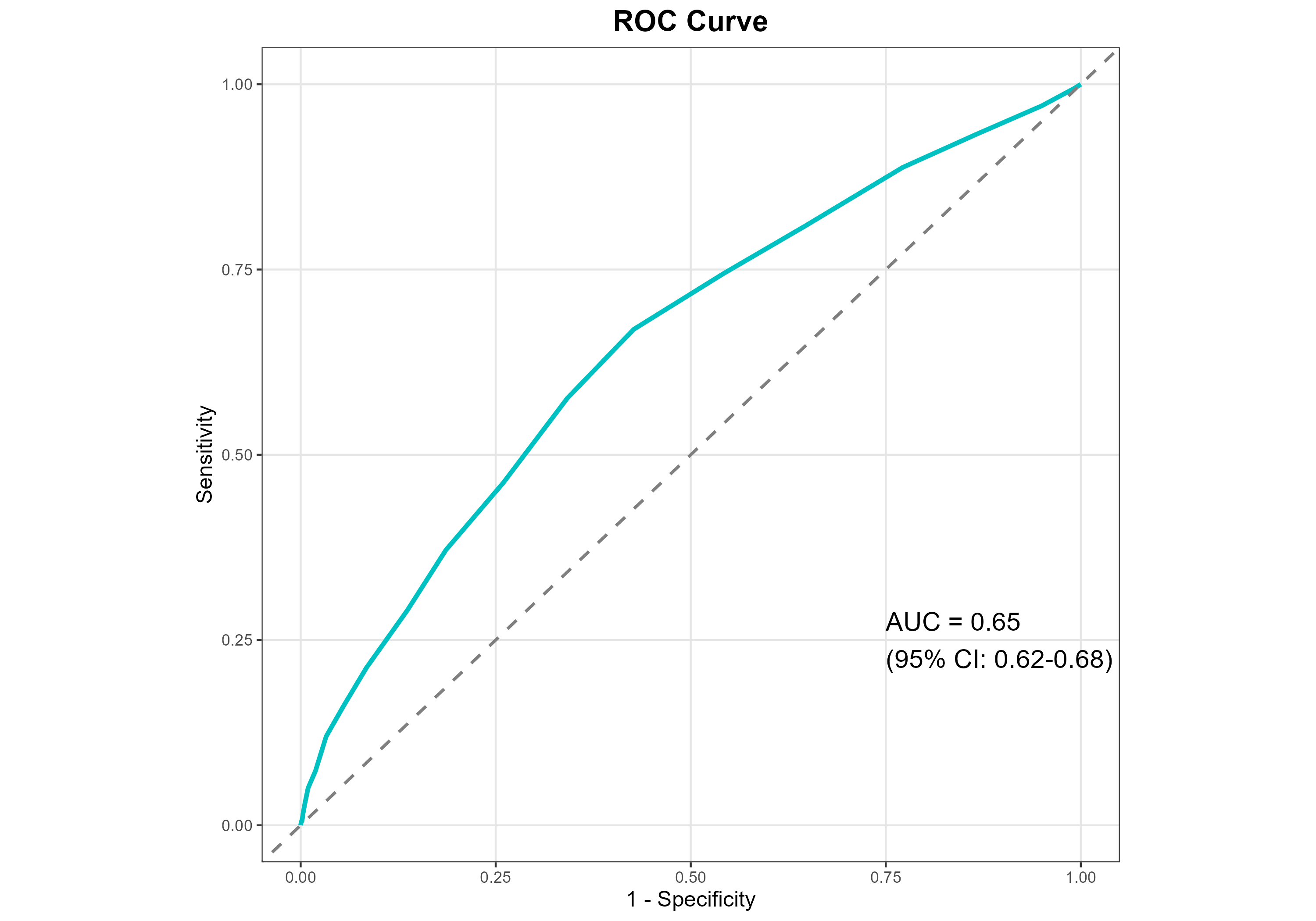

Supplement: Supplementary Figure 2 — ROC curve of the SOFA score alone for predicting in-hospital mortality in the MIMIC-IV validation cohort. [file Image2.tiff]

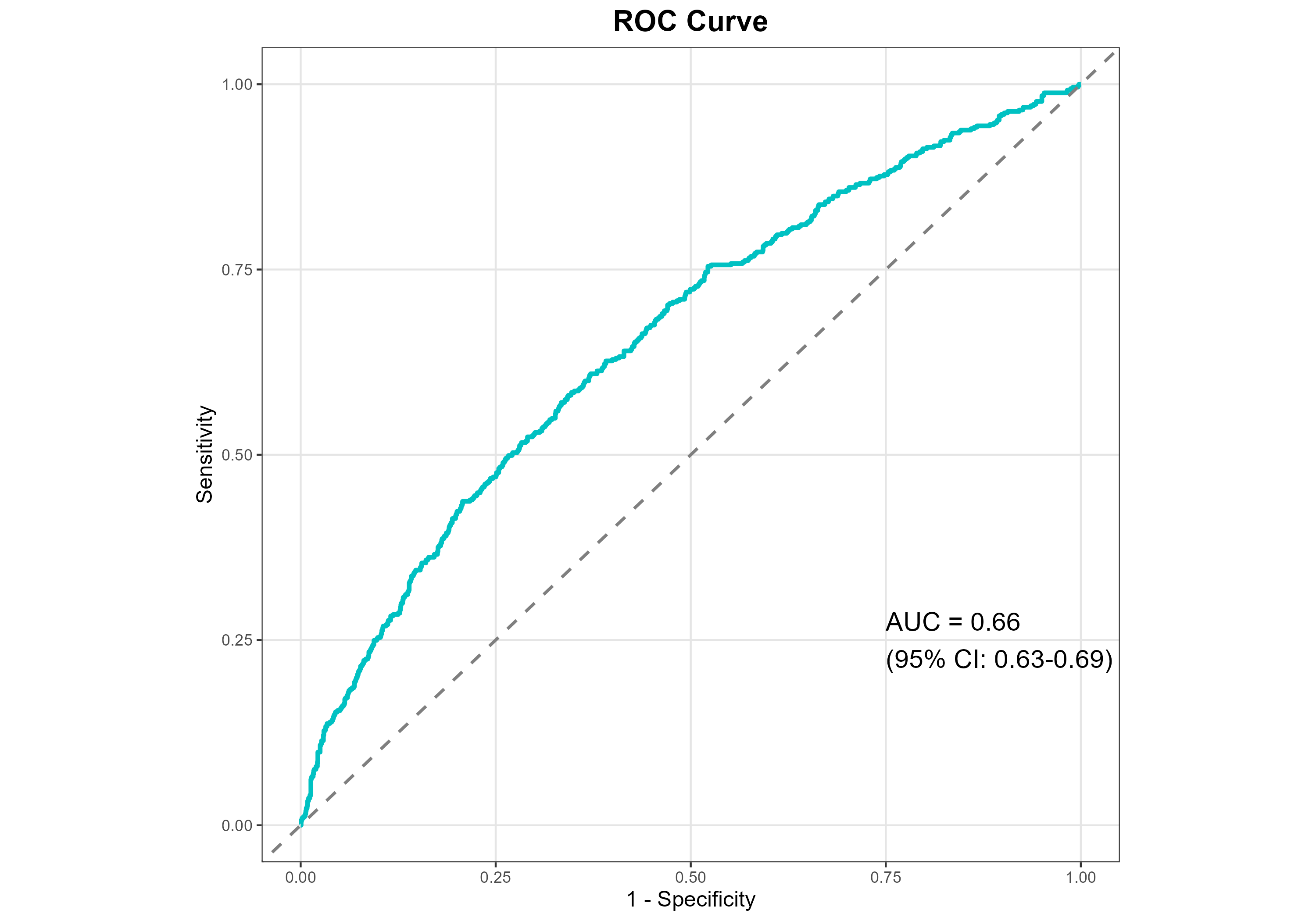

Supplement: Supplementary Figure 3 — ROC curve of the combination of the SOFA score and the CAR for predicting in-hospital mortality in the MIMIC-IV validation cohort. [file Image3.tiff]

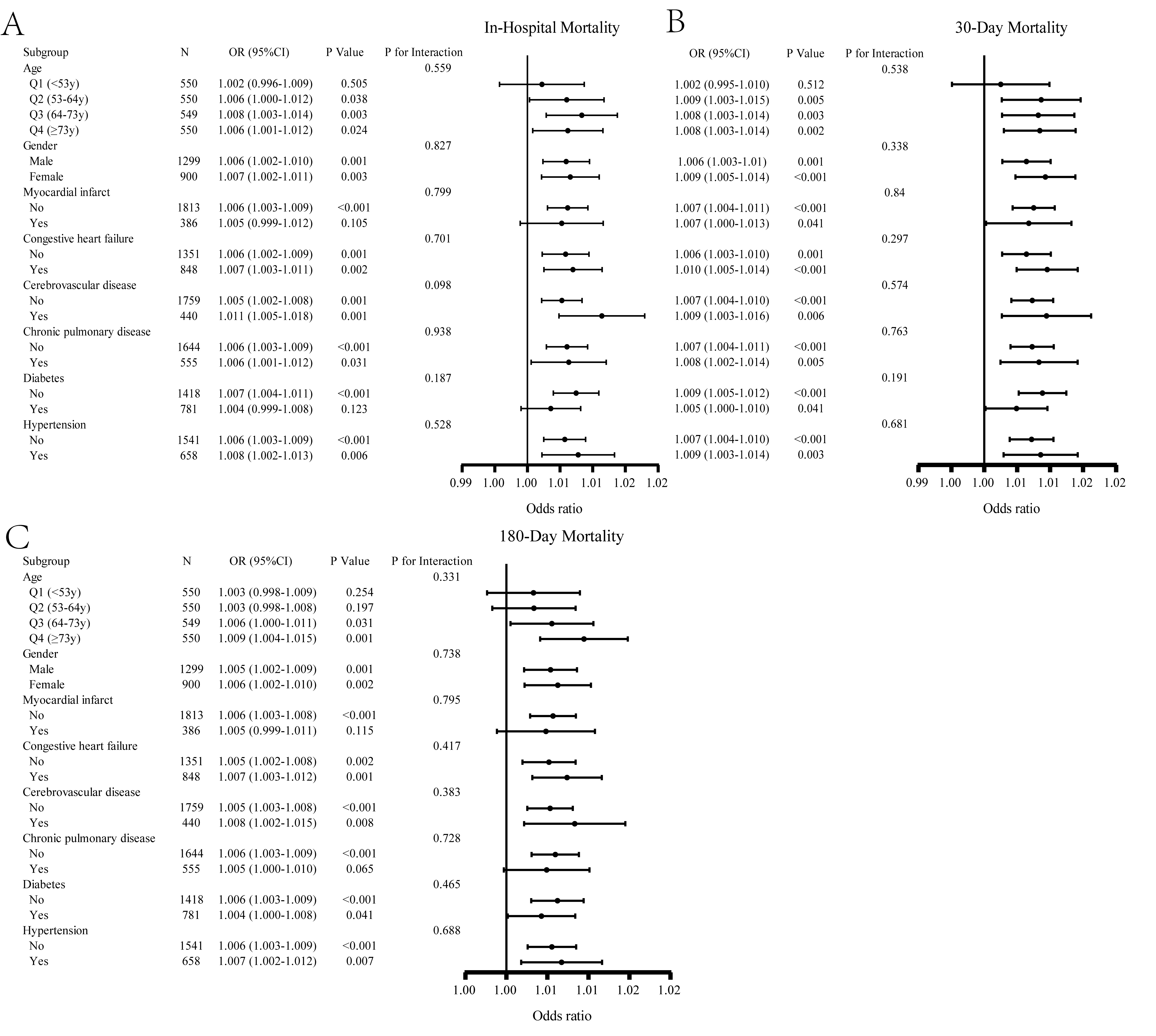

Supplement: Supplementary Figure 4 — Forest plots illustrating stratified analyses of association of CAR and all-cause mortality. (A) In-Hospital Mortality; (B) 30-Day Mortality; (C) 180-Day Mortality. [file Image4.tif]
