# Supplementary material for: Elevated C-reactive protein-to-albumin ratio as an independent prognostic marker for mortality in sepsis: a multicenter cohort study
Source: Front Cell Infect Microbiol. 2026 Jul 8;16:1772123. doi: 10.3389/fcimb.2026.1772123 (PMC13388168; doi:10.3389/fcimb.2026.1772123)
Supplement: Supplementary Table 1 — Baseline characteristics and outcomes of participants classified by CAR quartiles. [file Table2.docx]

**Table S2. Baseline characteristics of patients grouped based on In-Hospital Survival Status.**

| **Variables** | **Total** | **Survival (N=1682)** | **Non survival (N=517)** | **P value*** |
| --- | --- | --- | --- | --- |
| Female, n (%) | 900 (40.9%) | 679 (40.4%) | 221 (42.7%) | 0.357 |
| Age, y | 62.7 ± 15.4 | 61.4 ± 15.5 | 67.1 ± 14.5 | <0.001 |
| BMI, kg/m2 | 29.9 ± 8.6 | 29.7 ± 8.6 | 30.4 ± 8.7 | 0.036 |
| Comorbidities |  |  |  |  |
| Diabetes, n (%) | 781 (35.5%) | 580 (34.5%) | 201 (38.9%) | 0.072 |
| Hypertension, n (%) | 658 (29.9%) | 533 (31.7%) | 125 (24.2%) | <0.001 |
| Myocardial infarct, n (%) | 386 (17.6%) | 271 (16.1%) | 115 (22.2%) | <0.001 |
| Congestive heart failure, n (%) | 848 (38.6%) | 610 (36.3%) | 238 (46.0%) | <0.001 |
| Cerebrovascular disease, n (%) | 440 (20.0%) | 321 (19.1%) | 119 (23.0%) | 0.054 |
| Chronic pulmonary disease, n (%) | 555 (25.2%) | 419 (24.9%) | 136 (26.3%) | 0.530 |
| Vital signs |  |  |  |  |
| HR, beats/min | 88.6 ± 17.6 | 87.9 ± 17.2 | 90.8 ± 18.7 | 0.002 |
| RR, times/min | 21.1 ± 4.6 | 20.7 ± 4.5 | 22.4 ± 4.7 | <0.001 |
| SBP, mmHg | 114.5 ± 15.4 | 115.1 ± 15.7 | 112.4 ± 14.2 | 0.004 |
| DBP, mmHg | 62.5 ± 10.6 | 63.2 ± 10.7 | 60.4 ± 9.8 | <0.001 |
| MBP, mmHg | 77.6 ± 10.4 | 78.3 ± 10.6 | 75.6 ± 9.4 | <0.001 |
| Temperature, ℃ | 37.0 ± 0.6 | 37.0 ± 0.6 | 36.9 ± 0.7 | <0.001 |
| SpO2, % | 90.6 ± 7.4 | 91.1 ± 7.0 | 88.9 ± 8.4 | <0.001 |
| Laboratory parameters |  |  |  |  |
| Alb, g/dL | 3.0 ± 0.7 | 3.0 ± 0.7 | 2.8 ± 0.7 | <0.001 |
| CRP, mg/L | 106.1 ± 83.3 | 102.4 ± 82.4 | 117.9 ± 85.1 | <0.001 |
| Glucose, mg/dL | 113.2 ± 39.9 | 111.7 ± 37.7 | 118.1 ± 46.3 | 0.011 |
| WBC, 10^9^/L | 16.2 ± 12.1 | 15.7 ± 9.9 | 17.8 ± 17.2 | 0.028 |
| Hemoglobin, g/L | 9.6 ± 2.4 | 9.7 ± 2.3 | 9.4 ± 2.4 | 0.008 |
| Platelets, 10^9^/L | 200.5 ± 124.0 | 207.3 ± 123.2 | 178.2 ± 124.3 | <0.001 |
| Bun, mg/dL | 38.0 ± 29.3 | 35.2 ± 27.1 | 47.0 ± 34.0 | <0.001 |
| Hematocrit, % | 33.9 ± 6.8 | 34.1 ± 6.7 | 33.3 ± 7.3 | 0.001 |
| Creatinine, mg/24 h | 2.1 ± 2.1 | 2.1 ± 2.1 | 2.4 ± 2.0 | <0.001 |
| Anion gap, mEq/L | 13.0 ± 4.0 | 12.8 ± 3.8 | 13.8 ± 4.3 | <0.001 |
| Bicarbonate, mEq/L | 20.5 ± 5.3 | 20.6 ± 5.3 | 20.2 ± 5.5 | 0.128 |
| INR | 1.8 ± 1.4 | 1.7 ± 1.3 | 2.0 ± 1.5 | <0.001 |
| PT, s | 19.3 ± 13.9 | 18.7 ± 13.3 | 21.5 ± 15.5 | <0.001 |
| APTT, s | 48.2 ± 33.2 | 46.5 ± 31.9 | 53.6 ± 36.8 | <0.001 |
| SOFA | 6.8 ± 3.7 | 6.3 ± 3.5 | 8.4 ± 4.0 | <0.001 |
| Length of stay |  |  |  |  |
| Los ICU, day | 12.3 ± 13.7 | 12.2 ± 13.8 | 12.7 ± 13.2 | 0.016 |
| Los Hospital, day | 28.6 ± 27.1 | 30.1 ± 27.8 | 24.1 ± 24.4 | <0.001 |

*Statistically significant: a value less than 0.05 is interpreted as a meaningful difference

CAR, C-reactive protein to albumin ratio; BMI, Body Mass Index; HR, Heart rate; RR, Respiratory rate; SBP, Systolic blood pressure; DBP, Diastolic blood pressure; MBP, Mean blood pressure; SpO2, Peripheral capillary oxygen saturation; Alb, Albumin; CRP, C-reactive protein; WBC, White blood cells; Bun, Blood urea nitrogen; INR, International normalized ratio; PT, Prothrombin time; APTT, Activated partial thromboplastin time; SOFA, Sequential Organ Failure Assessment; Los, Length of Stay; ICU, Intensive care unit.
