# Supplementary material for: Elevated C-reactive protein-to-albumin ratio as an independent prognostic marker for mortality in sepsis: a multicenter cohort study
Source: Front Cell Infect Microbiol. 2026 Jul 8;16:1772123. doi: 10.3389/fcimb.2026.1772123 (PMC13388168; doi:10.3389/fcimb.2026.1772123)
Supplement: Supplementary Table 2 — Baseline characteristics of patients grouped based on In-Hospital Survival Status. [file Table1.docx]

**Table S1. Baseline characteristics and outcomes of participants classified by CAR quartiles.**

| **Variables** | **Total** | **Q1 (N=550, ≤12.02)** | **Q2 (N=550,**  **12.02-30.04)** | **Q3 (N=549, 30.04-60.15)** | **Q4 (N=550, ＞60.15)** | **P value*** |
| --- | --- | --- | --- | --- | --- | --- |
| Female, n (%) | 900 (40.9%) | 243 (44.2%) | 242 (44.0%) | 209 (38.1%) | 206 (37.5%) | 0.027 |
| Age, y | 62.7 ± 15.4 | 60.9 ± 15.6 | 63.2 ± 15.6 | 63.3 ± 15.6 | 63.5 ± 14.9 | 0.027 |
| BMI, kg/m2 | 29.9 ± 8.6 | 29.1 ± 8.0 | 29.4 ± 8.2 | 30.0 ± 9.0 | 31.0 ± 9.0 | <0.001 |
| Comorbidities |  |  |  |  |  |  |
| Diabetes, n (%) | 781 (35.5%) | 204 (37.1%) | 176 (32.0%) | 183 (33.3%) | 218 (39.6%) | 0.028 |
| Hypertension, n (%) | 658 (29.9%) | 148 (26.9%) | 171 (31.1%) | 167 (30.4%) | 172 (31.3%) | 0.333 |
| Myocardial infarct, n (%) | 386 (17.6%) | 75 (13.6%) | 106 (19.3%) | 100 (18.2%) | 105 (19.1%) | 0.048 |
| Congestive heart failure, n (%) | 848 (38.6%) | 212 (38.5%) | 217 (39.5%) | 216 (39.3%) | 203 (36.9%) | 0.818 |
| Cerebrovascular disease, n (%) | 440 (20.0%) | 133 (24.2%) | 115 (20.9%) | 108 (19.7%) | 84 (15.3%) | 0.003 |
| Chronic pulmonary disease, n (%) | 555 (25.2%) | 135 (24.5%) | 149 (27.1%) | 146 (26.6%) | 125 (22.7%) | 0.325 |
| Vital signs |  |  |  |  |  |  |
| HR, beats/min | 88.6 ± 17.6 | 87.5 ± 17.2 | 87.1 ± 18.1 | 88.5 ± 17.7 | 91.2 ± 17.1 | <0.001 |
| RR, times/min | 21.1 ± 4.6 | 19.9 ± 4.3 | 20.5 ± 4.2 | 21.5 ± 4.7 | 22.5 ± 4.8 | <0.001 |
| SBP, mmHg | 114.5 ± 15.4 | 116.6 ± 15.8 | 114.7 ± 15.8 | 113.7 ± 15.1 | 112.9 ± 14.7 | 0.002 |
| DBP, mmHg | 62.5 ± 10.6 | 64.1 ± 11.1 | 62.8 ± 10.9 | 61.8 ± 10.3 | 61.3 ± 9.7 | <0.001 |
| MBP, mmHg | 77.6 ± 10.4 | 79.1 ± 10.9 | 78.0 ± 10.7 | 77.0 ± 10.0 | 76.4 ± 9.8 | <0.001 |
| Temperature, ℃ | 37.0 ± 0.6 | 36.9 ± 0.6 | 36.9 ± 0.6 | 37.0 ± 0.6 | 37.1 ± 0.7 | <0.001 |
| SpO2, % | 90.6 ± 7.4 | 91.2 ± 7.8 | 91.2 ± 7.3 | 90.0 ± 7.6 | 89.9 ± 6.9 | <0.001 |
| Laboratory parameters |  |  |  |  |  |  |
| Alb, g/dL | 3.0 ± 0.7 | 3.3 ± 0.6 | 3.1 ± 0.6 | 2.9 ± 0.6 | 2.6 ± 0.6 | <0.001 |
| CRP, mg/L | 106.1 ± 83.3 | 15.6 ± 11.8 | 62.6 ± 19.4 | 126.3 ± 37.6 | 219.7 ± 48.9 | <0.001 |
| Glucose, mg/dL | 113.2 ± 39.9 | 110.6 ± 39.5 | 111.5 ± 36.9 | 113.0 ± 38.7 | 117.8 ± 44.0 | 0.010 |
| WBC, 10^9^/L | 16.2 ± 12.1 | 14.4 ± 9.1 | 16.0 ± 9.9 | 16.9 ± 15.3 | 17.5 ± 12.9 | <0.001 |
| Hemoglobin, g/L | 9.6 ± 2.4 | 9.7 ± 2.5 | 9.5 ± 2.2 | 9.6 ± 2.3 | 9.6 ± 2.4 | 0.586 |
| Platelets, 10^9^/L | 200.5 ± 124.0 | 185.8 ± 124.5 | 201.8 ± 114.7 | 200.9 ± 121.2 | 213.4 ± 133.7 | <0.001 |
| Bun, mg/dL | 38.0 ± 29.3 | 34.7 ± 28.5 | 37.0 ± 28.2 | 38.4 ± 30.0 | 41.8 ± 30.0 | <0.001 |
| Hematocrit, % | 33.9 ± 6.8 | 34.3 ± 6.9 | 33.9 ± 6.5 | 33.7 ± 7.0 | 33.8 ± 7.0 | 0.343 |
| Creatinine, mg/24 h | 2.1 ± 2.1 | 1.9 ± 1.9 | 2.0 ± 1.9 | 2.2 ± 2.1 | 2.4 ± 2.4 | <0.001 |
| Anion gap, mEq/L | 13.0 ± 4.0 | 12.6 ± 3.8 | 12.8 ± 4.2 | 13.2 ± 3.9 | 13.4 ± 3.9 | <0.001 |
| Bicarbonate, mEq/L | 20.5 ± 5.3 | 20.8 ± 5.5 | 20.5 ± 5.5 | 20.4 ± 5.0 | 20.4 ± 5.3 | 0.190 |
| INR | 1.8 ± 1.4 | 1.7 ± 1.0 | 1.8 ± 1.4 | 1.8 ± 1.5 | 1.8 ± 1.5 | 0.005 |
| PT, s | 19.3 ± 13.9 | 18.4 ± 10.7 | 20.0 ± 15.6 | 19.4 ± 13.9 | 19.5 ± 14.9 | 0.011 |
| APTT, s | 48.2 ± 33.2 | 46.6 ± 31.6 | 47.1 ± 32.6 | 48.9 ± 34.9 | 50.2 ± 33.7 | 0.031 |
| SOFA | 6.8 ± 3.7 | 6.4 ± 3.8 | 6.6 ± 3.7 | 7.0 ± 3.6 | 7.3 ± 3.7 | <0.001 |
| Length of stay |  |  |  |  |  |  |
| Los ICU, day | 12.3 ± 13.7 | 11.3 ± 14.5 | 11.6 ± 12.5 | 13.0 ± 14.2 | 13.4 ± 13.2 | <0.001 |
| Los Hospital, day | 28.6 ± 27.1 | 29.4 ± 28.4 | 29.0 ± 32.8 | 28.4 ± 23.0 | 27.8 ± 23.2 | 0.782 |
| Clinical outcomes |  |  |  |  |  |  |
| In-Hospital Mortality, n (%) | 517 (23.5%) | 109 (19.8%) | 111 (20.2%) | 142 (25.9%) | 155 (28.2%) | 0.002 |
| 30-Day Mortality, n (%) | 459 (20.9%) | 96 (17.5%) | 94 (17.1%) | 120 (21.9%) | 149 (27.1%) | <0.001 |
| 180-Day Mortality, n (%) | 806 (36.7%) | 176 (32.0%) | 185 (33.6%) | 212 (38.6%) | 233 (42.4%) | 0.001 |

*Statistically significant: a value less than 0.05 is interpreted as a meaningful difference

CAR, C-reactive protein to albumin ratio; BMI, Body Mass Index; HR, Heart rate; RR, Respiratory rate; SBP, Systolic blood pressure; DBP, Diastolic blood pressure; MBP, Mean blood pressure; SpO2, Peripheral capillary oxygen saturation; Alb, Albumin; CRP, C-reactive protein; WBC, White blood cells; Bun, Blood urea nitrogen; INR, International normalized ratio; PT, Prothrombin time; APTT, Activated partial thromboplastin time; SOFA, Sequential Organ Failure Assessment; Los, Length of Stay; ICU, Intensive care unit.
