# Supplementary material for: Elevated C-reactive protein-to-albumin ratio as an independent prognostic marker for mortality in sepsis: a multicenter cohort study
Source: Front Cell Infect Microbiol. 2026 Jul 8;16:1772123. doi: 10.3389/fcimb.2026.1772123 (PMC13388168; doi:10.3389/fcimb.2026.1772123)
Supplement: Supplementary Table 3 — Multivariate Cox Proportional Hazards Analysis of CAR Quartiles and Mortality. [file Table3.docx]

**Table S3. Multivariate Cox Proportional Hazards Analysis of CAR Quartiles and Mortality.**

| Categories | Model 1 | | | Model 2 | | | Model 3 | | |
| --- | --- | --- | --- | --- | --- | --- | --- | --- | --- |
|  | HR (95%CI) | P value | P for trend | HR (96%CI) | P value | P for trend | HR (97%CI) | P value | P for trend |
| **In-Hospital Mortality** |  |  |  |  |  |  |  |  |  |
| Quartile^*^ |  |  | **< 0.001** |  |  | **< 0.001** |  |  | **0.006** |
| Q1 | 1 (Ref.) |  |  | 1 (Ref.) |  |  | 1 (Ref.) |  |  |
| Q2 | 1.04 (0.80-1.35) | 0.779 |  | 0.99 (0.76-1.29) | 0.926 |  | 1.01 (0.77-1.32) | 0.959 |  |
| Q3 | 1.37 (1.06-1.76) | 0.014 |  | 1.29 (1.00-1.65) | 0.049 |  | 1.21 (0.94-1.57) | 0.145 |  |
| Q4 | 1.56 (1.22-2.00) | < 0.001 |  | 1.47 (1.15-1.88) | 0.002 |  | 1.38 (1.06-1.79) | 0.016 |  |
| **30-Day Mortality** |  |  |  |  |  |  |  |  |  |
| Quartile |  |  | **< 0.001** |  |  | **< 0.001** |  |  | **< 0.001** |
| Q1 | 1 (Ref.) |  |  | 1 (Ref.) |  |  | 1 (Ref.) |  |  |
| Q2 | 0.99 (0.75-1.32) | 0.957 |  | 0.93 (0.70-1.24) | 0.624 |  | 0.96 (0.72-1.28) | 0.78 |  |
| Q3 | 1.29 (0.99-1.69) | 0.059 |  | 1.21 (0.92-1.58) | 0.172 |  | 1.17 (0.89-1.54) | 0.267 |  |
| Q4 | 1.70 (1.31-2.19) | < 0.001 |  | 1.59 (1.23-2.06) | < 0.001 |  | 1.55 (1.18-2.04) | 0.002 |  |
| **180-Day Mortality** |  |  |  |  |  |  |  |  |  |
| Quartile |  |  | **< 0.001** |  |  | **< 0.001** |  |  | **< 0.001** |
| Q1 | 1 (Ref.) |  |  | 1 (Ref.) |  |  | 1 (Ref.) |  |  |
| Q2 | 1.07 (0.87-1.31) | 0.537 |  | 1.00 (0.81-1.23) | 0.993 |  | 1.03 (0.83-1.27) | 0.788 |  |
| Q3 | 1.27 (1.04-1.55) | 0.019 |  | 1.20 (0.98-1.47) | 0.076 |  | 1.20 (0.98-1.47) | 0.082 |  |
| Q4 | 1.49 (1.22-1.81) | < 0.001 |  | 1.43 (1.18-1.74) | < 0.001 |  | 1.42 (1.16-1.75) | 0.001 |  |

Model 1: unadjusted

Model 2: adjusted for Age, Gender, BMI

Model 3: adjusted for Age, Gender, BMI, Myocardial infarct, Congestive heart failure, Cerebrovascular disease, Chronic pulmonary disease, Hypertension, diabetes, HR, RR, SBP, DBP, Temperature, SpO2, WBC, Hemoglobin, Platelets, Bun, Calcium, Hematocrit, Creatinine, Sodium, Potassium, Anion gap, Bicarbonate, Chloride, INR, PT, APTT

^*^CAR Quartile: Q1 (≤12.02), Q2 (12.02-30.04), Q3 (30.04-60.15), Q4 (＞60.15).
